# Accommodations for Patients with Disabilities in Primary Care: A Mixed Methods Study of Practice Administrators

**DOI:** 10.5539/gjhs.v6n1p23

**Published:** 2013-10-08

**Authors:** Jennifer R Pharr

**Affiliations:** 1 University of Nevada, Las Vegas, United States

**Keywords:** people with disabilities, accommodations, primary care, health care access, Americans with disabilities Act

## Abstract

Structural barriers that limit access to health care services for people with disabilities have been identified through qualitative studies; however, little is known about how patients with disabilities are accommodated in the clinical setting when a structural barrier is encountered. *The purpose of this* study was to identify how primary care medical practices in the United States accommodated people with disabilities when a barrier to service is encountered. Primary care practice administrators from the medical management organization were identified through the organization’s website. Sixty-three administrators from across the US participated in this study. Practice administrators reported that patients were examined in their wheelchairs (76%), that parts of the exam where skipped when a barrier was encountered (44%), that patients were asked to bring someone with them (52.4%) or that patients were refused treatment due to an inaccessible clinic (3.2%). These methods of accommodation would *not* be in compliance with requirements of the Americans with Disabilities Act. There was not a significant difference (p>0.05) in accommodations for patients with disabilities between administrators who could describe the application of the ADA to their clinic and those who could not. Practice administrators need a comprehensive understanding of the array of challenges encountered by patients with disabilities throughout the health care process and of how to best accommodate patients with disabilities in their practice.

## 1. Introduction

### 1.1 Importance of the Problem

People with disabilities experience unmet health care needs which lead to health disparities. Health disparity has been defined by a number of authors and organizations (Braveman, 2006; Carter-Pokras & Baquet, 2002; Kilbourne, Switzer, Hyman, Crowley-Matoka, & Fine, 2006; National Institute of Health, 2000; Nelson, 2002). In its simplest form, health disparities can be described as the preventable, population-specific differences in rates of disease, quality of health care or access to health care (Health Resources and Services Administration, 2000). People with disabilities in the United States are more likely to have chronic diseases and secondary conditions and less likely to engage in some preventive health services such as dental cleanings, mammograms, breast exams and Pap tests (Armour, Thierry, & Wolf, 2009; Chan et al., 1999; Chang & Krosnick, 2010; Cheng et al., 2001; Diab & Johnston, 2004; Havercamp, Scandlin, & Roth, 2004; Iezzoni, McCarthy, Davis, & Siebens, 2000; Iezzoni, McCarthy, Davis, Harris-David, & O'Day, 2001; Iezzoni, Park, & Kilbridge, 2011; Nosek & Howland, 1997; Pharr & Moonie, 2011; Ramirez, Farmer, Grant, & Papachristou, 2005; Reichard, Stolzle, & Fox, 2011; Thierry, 2000; Wei, Findley, & Sambamoorthi, 2006).

In 1990, The Americans with Disabilities Act (ADA) became a federal civil rights law in the US. This law prohibits discrimination against people with disabilities in both public and private places of business. Legally, people with disabilities must be granted equal access to buildings and services (Americans with Disabilities Act, 2008). The ADA applies not only to commercial places of business but to public and private health care facilities as well. However, there are many barriers that keep people with disabilities from fully engaging in health care and lead to disparities in the delivery of preventive health services (Barr, Giannotti, Van Hoof, Mongoven, & Curry, 2008; Becker, Stuifbergen, & Tinkle, 1997; Drainoni et al., 2006; Kroll, Jones, Kehn, & Neri, 2006; Mele, Archer, & Pusch, 2005; Scheer, Kroll, Neri, & Beatty, 2003; Story, Schwier, & Kailes, 2009).

Several qualitative research studies have been conducted with people with disabilities to understand why they experience unmet healthcare needs (Barr et al., 2008; Becker et al., 1997; Center for Disease Control and Prevention, 2006; Drainoni et al., 2006; Mele et al., 2005; Scheer et al., 2003; Story et al., 2009). Three main categories of barriers were identified and include structural, financial and personal/cultural barriers *(Drainoni et al., 2006)*. Of interest for this study are structural barriers that people with disabilities encounter when accessing health care services. These include: inadequate disability parking, lack of ramps or ramps with too steep of a grade, narrow doorways, heavy doors without automatic opening capabilities, lack of elevators, cramped waiting rooms, examination rooms that are too small in which to maneuver a wheelchair, scales that cannot accommodate a wheelchair, examination tables that are not height adjustable, inaccessible diagnostic equipment and inaccessible restrooms (Becker et al., 1997; Kroll et al., 2006; Mele et al., 2005; Scheer et al., 2003; Story et al., 2009).

### 1.2 Purpose of the Study

Although structural barriers that limit access to health care services for people with disabilities have been identified through qualitative interviews, little is known about how patients with disabilities are accommodated in the clinical setting when a barrier to service is encountered. This presents a gap in knowledge concerning the unmet health care needs and resulting health disparities that people with disabilities experience. *The purpose of this* mixed methods study was to identify how primary care clinics in the United States accommodated people with disabilities when a structural barrier was encountered. A goal of this study was to contribute to the body of literature regarding health disparities of people with disabilities and to provide data that might be used to shape disability policy in the United States.

## 2. Methods and Materials

### 2.1 Study Design

A convergent mixed methods research design was used because this study explored both quantitative and qualitative concepts. This design allowed for qualitative and quantitative data to be collected simultaneously from a single population (Creswell & Clark, 2007). Primary care practice administrators who were members of a medical management organization were the population selected for this study. Primary care clinics were chosen for this study because the majority of preventive care occurs in primary care clinics (Harrington, Hirsch, Hammond, Norton, & Bockenek, 2009). Primary care clinics included general practice clinics, family practice clinics, internal medicine and obstetrics/gynecology clinics. Practice administrators were selected for this study because their position usually has oversight of facility operations and patient flow and accommodations for patients with disabilities would be within their purview (Handbook, 2004). IRB approval was obtained prior to data collection.

### 2.2 Study Instrument

The survey used for this study was developed using ADA construction guidelines, the ADA’s *Access to Medical Care for Individuals with Mobility Disabilities* and published literature (Adaptive Environment Center, 1995; Architectural, 1991; Grabois, Nosek, & Rossi, 1999; Graham & Mann, 2008; Harrington et al., 2009; Sanchez et al., 2000; U.S. Department of Justice & U.S. Department of Health and Human Rights, 2010). The survey included demographic questions, questions regarding the administrator’s knowledge of the ADA and questions about how patients with disabilities were accommodated when a barrier to care was encountered. ADA knowledge questions were qualitative in nature and included: Can you, briefly describe the purpose of the ADA as you understand it? Are you aware that the ADA applies to medical practices? If yes, describe how the ADA applies to medical practices?

### 2.3 Participants

Primary care practice administrators from across the United States who were members of a medical management organization were identified through the organization’s website. Practice administrators who self-identified as primary care administrators were contacted through the website e-group communication portal. In total, 1,637 practice administrators were sent a message through the e-group communication system on three separate dates between December 20, 2011 and January 17, 2012. Eighty-six administrators initiated the survey through the survey link. Of those who initiated the survey, sixty-three completed the survey for a completion rate of 73.3%. The number of administrators who viewed the message and refused to participate or the number of administrators who did not view the message (non-contact) could not be determined using the communication portal. Because of this, it was not possible to calculate an accurate contact, cooperation or response rate.

### 2.4 Statistical Analysis

Data were analyzed in 2012. Data were prepared for analysis and analyzed by separate means, either quantitative (statistical software) or qualitative (coding and development of themes). Descriptive statistics were computed for the group (Table 1). Qualitative data from ADA knowledge questions were analyzed for major themes. Proportions of accommodation methods were calculated (Table 2). Chi square analyses were conducted to determine if there was a difference in accommodations between administrators who could describe how the ADA applied to their practice and administrators who could not.

**Table 1:** Descriptive characteristics of administrators and practices

	Practice Administrators n = 63	
Variable	Mean	SD
Age	49.6	8.3
Years as administrator	14.9	9.3
Years at current practice	5.9	4.7
Years practice in operation	27	18.6
Number of providers	12.7	12.2
Number of patients	29,561	48,470
% Patients with disabilities	7.8	14.6

Variable	n	%
Gender		
Female	44	69.8
Male	18	28.6
Education		
High school	1	1.6
Associate’s Degree	4	6.3
Bachelor’s Degree	22	34.9
Master’s Degree	30	47.6
Doctoral Degree	0	0
Other - Professional	6	9.5
Type of Practice		
Family Medicine	15	23.8
Internal Medicine	10	15.9
General Medicine	1	1.6
OB/GYN	30	47.6
Other	7	11.1
Building Built		
Before 1993	32	50.8
After 1993	30	47.6

**Table 2:** Accommodations for patients with disabilities

	Practice Administrators n = 63	
Variable	n	%
Patient is examined in their wheelchair	48	76.2
Employees trained to lift patient	49	77.8
Patient asked to bring someone with them to help transfer	33	52.4
Lift available	3	4.8
Patient is referred to another clinic	30	47.6
That part of the exam is skipped	28	44.4
Refused treatment because:		
Practice was inaccessible	2	3.2
It took longer to examine them	0	0
Practice not reimbursed for longer exam	0	0

## 3. Results

### 3.1 Descriptive Characteristics of the Administrators

Descriptive characteristics of the administrators and practices are provided in Table 1.

### 3.2 Qualitative Analysis Results

Practice administrators were asked: Can you briefly describe the purpose of the ADA as you understand it? Four themes emerged from this question. Three of the themes were consistent with a general explanation of the ADA: 1) to eliminate discrimination, 2) provide accommodations and 3) ensure accessibility. The fourth theme to emerge was an inability to describe the ADA. Sample responses included:

*Respondent 4: Civil rights law that does not allow discrimination based upon disability*. (December, 2011)

*Respondent 36: To make sure that people with physical disabilities are afforded the same accessibility that non-disabled patients are. To remove all barriers to access*. (December, 2011)

*Respondent 52: To provide reasonable accommodations for people with physical or mental disabilities*. (January, 2012)

Practice administrators who knew that the ADA applied to medical practices were asked to describe how the ADA applies to medical practices? The four themes that emerged from this question included: 1) accommodations for patients with disabilities (PWD), 2) accessibility for PWD, 3) access to employment for people with disabilities, and 4) not being able to describe. Example responses were:

*Respondent 25: Patients must be able to access healthcare facilities and receive health care that is appropriate to their needs. Facilities must be easily accessible*. (December, 2011)

*Respondent 30: That all resources, including policies and procedures, accommodate to all persons, regardless of handicap and that reasonable accommodation be made to ensure that access*. (December, 2011)

*Respondent 27: Making our facility accessible to employees and patients with disabilities without assistance*. (December, 2011)

### 3.3 Accommodations for Patients with Disabilities

Accommodation results are provided in table 2. Administrators were asked if patients were referred to another practice if a barrier to services was encountered at their practice. Forty-seven percent of the administrators responded ‘yes’ to this question. Administrators were asked if parts of an exam were skipped when a barrier to service was encountered when examining a patient with disabilities. Forty-four percent of the administrators acknowledged that parts of an exam were skipped when a barrier was encountered. Practice administrators were asked what alternatives were used if a patient was not able to transfer onto an exam table. Seventy-six percent of practice administrators indicated that patients were examined in their wheelchairs when they cannot transfer onto an exam table. 52.4% of practice administrators reported asking patients to bring someone with them to help with the transfer. Seventy-seven percent of practice administrators indicated that their employees were trained to lift a patient while 4.8% of practices have a lift available to transfer patients.

Chi square tests were conducted to determine if there was a difference in accommodations between administrators who could describe how the ADA applied to their practice and administrators who could not. No significant differences (p > 0.05) were found between groups with regard to skipping part of the exam, examining patients in their wheelchairs, asking patients to bring someone with them, referring patients to another practice, having employees trained to assist patients or having a lift.

## 4. Discussion

### 4.1 Accommodations for People with Disabilities

The most interesting finding from this study was the number of administrators who reported that: part of an exam was skipped if a barrier was encountered, patients were examined in their wheelchairs if they cannot transfer onto the exam table, or patients were asked to bring someone with them to assist. Based on Title II and Title III of the ADA, services offered at a medical practice must be fully accessible to patients with disabilities (U.S. Department of Justice & U.S. Department of Health and Human Rights, 2010). For the most part, these three methods of accommodating patients with disabilities are not in compliance with ADA guidelines. In this study, nearly half of the administrators acknowledge that part of an exam was skipped when a barrier was encountered. This finding supports previous research which has shown that patients with disabilities are less likely to engage in some preventive services (Armour et al., 2009; Chan et al., 1999; Cheng et al., 2001; Diab & Johnston, 2004;Havercamp et al., 2004; Iezzoni et al., 2000; Kroll et al., 2006; Mele et al., 2005; Pharr & Moonie, 2011; Reichard et al., 2011). For example, women with disabilities are less likely to have a Pap test, mammogram or breast exam in part due to a lack of height adjustable exam tables or accessible mammography equipment. In a qualitative study of both patients with disabilities and family physicians, both patients and physicians acknowledged that physicians did not have the time, training or equipment necessary to perform a complete physical exam on patients with disabilities (Morrison, George, & Mosqueda, 2008). However, the ADA’s requirement of full and equal access to all services would imply that if a patient without disabilities receives a complete physical examination, then a patient with disabilities should be afforded the same care. Patients with disabilities should receive medical services that are equal to those received by patients without disabilities (U.S. Department of Justice & U.S. Department of Health and Human Rights, 2010). The same standard of care must apply to all patients.

A large majority of administrators acknowledged that patients were examined in their wheelchair which is also consistent with previous research (Grabois et al., 1999; Mele et al., 2005). In surveying primary care physicians, Grabois et al. found that over half of physicians believed they could perform an adequate examination with a patient seated in his/her wheelchair 35. In general, this would not be an acceptable accommodation in accordance with the ADA. For many conditions, examining patients in their wheelchairs is a less thorough than examining them on an exam table (U.S. Department of Justice & U.S. Department of Health and Human Rights, 2010). Some examinations, such as an ear/nose/throat exam, might be accomplished with equal quality whether a patient is seated in a wheelchair or on an exam table (Bickley, Szilagyi, & Bates, 2008; Seidel, Ball, Dains, & Benedict, 2006). However, standard protocol would dictate that palpation of the abdomen or liver, a gynecological examination, or an EKG be performed with a patient in a supine position (Bickley et al., 2008; Fischbach & Dunning, 2009; Seidel et al., 2006). To perform these exams with a patient seated in a wheelchair would result in a lower quality of care and lead to a disparity in the receipt of health care services between those with and without a disability.

Over half of the administrators reported that patients are asked to bring someone with them to help at the exam. The ADA requires that medical practices provide reasonable assistance so patients with disabilities can receive care (U.S. Department of Justice & U.S. Department of Health and Human Rights, 2010). Patients with disabilities, just as patients without disabilities, may choose to bring someone with them to their medical appointment. They may also choose to go to medical appointments alone. Medical practices cannot ask a patient with disabilities to bring someone with them, although this study and previous studies have found that this does occur (Morrison et al., 2008). Findings that primary care practices skip parts of examinations, examine patients in their wheelchairs or ask patients with disabilities to bring someone with them support the need for a better understanding of the ADA’s requirement of *fully and equal access* to medical services on the part of health care administrators and physicians.

There are additional ways of accommodating patients with disabilities when barriers to service are encountered which are consistent with the ADA guidelines. These include having employees trained to assist patients or providing a lift to help the patient transfer onto an exam table (U.S. Department of Justice & U.S. Department of Health and Human Rights, 2010). Seventy-seven percent of the administrators reported that their employees were trained to lift patients. Although it is important to provide training for employees regarding proper lifting techniques, lifting a patient onto an exam table creates a safety concern for health care employee as well as the patient. Back injuries are common among health care workers and are caused mainly from transferring patients. A study by Hart in 2006 found thirty-eight percent of nurses and forty-two percent of radiology technicians had experienced an injury due to moving, lifting or repositioning a patient in a two year time period (Hart, 2006). Studies have also found that patients have been injured by health care workers who are not trained in proper lifting techniques or from falling off of examine tables that are too high (Kirschner, Breslin, & Iezzoni, 2007). Five percent of the administrators reported that there were lifts available to transfer a patient onto an exam table. The low percentage of lifts identified in this study is concerning because a lift can help prevent health care employees and patients from injury (U.S. Department of Justice & U.S. Department of Health and Human Rights, 2010).

An analysis was performed to determine if there was a difference in accommodations between administrators who could or could not describe how the ADA applied to their clinics. No significant difference was found between the groups. Clinics that had administrators who could describe how the ADA applied to their clinics were equally likely to skip parts of an exam, examine patients in their wheelchairs or ask patients to bring someone with them. When administrators were asked to describe the ADA in general and to describe how the ADA applied to their medical practice, they used statement such as: “completely accessible”, “reasonable accommodations”, “full access”, “non-discrimination”, “full and equal enjoyment of goods and services”, “to make sure that people with physical disabilities are afforded the same accessibility that non-disabled patients are”. However, these administrators also reported that: parts of exam were skipped, patients were asked to bring someone with them, or patients were examined in their wheelchairs. This finding represents a disconnect between what the administrators said purpose of the ADA was and how they accommodated patients with disabilities when a barrier was encountered. The ADA requires that patients with disabilities have equal access to all services. This means more than just ramps into a clinic (Reis, Breslin, Iezzoni, & Kirschner, 2004). Patients with disabilities need to be able to fully participate in all health care services offered, especially preventive care. Practices administrators need a comprehensive understanding of the array of challenges encountered by patients with disabilities when accessing preventive health care and of appropriate ways to accommodate them.

When considering the ADA’s requirement that patients with disabilities have ‘full and equal access to health care facilities and services’ it may be easier for practices to be compliant with access to facilities than access to service (U.S. Department of Justice & U.S. Department of Health and Human Rights, 2010). Unambiguous guidelines for building construction and renovation are provided in the ADA’s *Accessibility Guidelines for Buildings and Facilities*. For example, doors must have at least a 32” clear opening; buildings with stairs must have a wheelchair accessible elevator, a lift or a ramp; or door handles should be no higher than 48” and operable with a closed fist (Adaptive Environment Center, 1995). However, equal access to services might be a more difficult concept to understand, especially for those who have not received disability education. Previous research has found that knowledge of the ADA is a key to reducing the number of barriers to health care and that the number of pieces of accessible equipment is positively correlated with knowledge of accessible equipment among health care administrators (Pharr & Chino, 2013; Pharr, 2013). However, few educational programs exist that provide disability training for health professionals, particularly health care administrators (Iezzoni, Ramanan, & Drews, 2005; Kroll, Beatty, & Bingham, 2003; McNeal, Carrothers, & Premo, 2002; Shakespeare, Iezzoni, & Groce, 2009; Tervo, Azuma, Palmer, & Redinius, 2002; Veltman, Stewart, Tardif, & Branigan, 2001). Health care professionals who have little or no disability training may not be aware of how to best accommodate their patients with disabilities (Yee & Breslin, 2010).

To help educate health care professionals about access to health care and accommodations for patients with disabilities, the U.S Department of Justice released *Access to Medical Care for Individuals with Mobility Disabilities* in 2010 (U.S. Department of Justice & U.S. Department of Health and Human Rights, 2010). This document was intended to provide health care professionals with information regarding how the ADA applies to their practice. The document includes an overview of the ADA and general requirements for medical practices. In the section of commonly asked questions there is information about ways to accommodate patients that are in compliance with the ADA and information about ways that are not in compliance with the ADA. This document is a valuable resource for health care providers and administrators.

### 4.2 Limitations

Limitations have been identified in this study. There was a possibility of bias resulting from self-reported information. The participants may have under or over reported information if they perceived the response to be socially desirable (Adams, Soumerai, Lomas, & Ross-Degnan, 1999). Although we could not calculate an accurate response rate, a low percentage of administrators participated in this study compared to the number invited to participate. Studies with low response rates are susceptible to self-selection bias (Aschengrau & Seage, 2003). Although the sample size for mixed methods research tends to be lower than purely quantitative research, the sample size for this study was relatively low for the quantitative analysis portion of the study (Creswell & Clark, 2007).

The current study only focused on primary care. Results cannot be generalized to specialty practices such as cardiology or oncology. However the practice administrators were from across the United States making the results more generalizable to primary care practices. The current study only included practice administrators. Clinical staff, such as nurses or physicians, may have had better insight into how patients with disabilities are accommodated in their clinics (Sanchez et al., 2000).

## 5. Conclusion

Despite the potential limitations, important discoveries were made. This was the first study to specifically address ways that patients with disabilities are accommodated in primary care clinics in the US through survey of practice administrators. The administrators reported that patients with disabilities were examined in their wheelchairs, were asked to bring someone with them or that part of an examination was skipped if a barrier was encountered. These findings highlight the need for improved ADA and disabilities knowledge among primary care practice administrators. Including ADA and disability education into health care administrator college curricula or continuing medical education courses may be a means to increase administrators’ knowledge of the ADA and how to best accommodate their patients with disabilities.
